# Amplicon sequence collection of putative polyethylene terephthalate hydrolases from two different composts in Japan

**DOI:** 10.1128/mra.00173-26

**Published:** 2026-04-20

**Authors:** Ryo Iizuka, Toshiyuki Moriya, Tairo Oshima, Sotaro Uemura, Masafumi Yohda

**Affiliations:** 1 Department of Biological Sciences, Graduate School of Science, The University of Tokyo 13143, Tokyo, Japan; 2 Institute of Environmental Biology, Kyowa-Kako, Tokyo, Japan; 3 Core Research for Evolutional Science and Technology (CREST), Japan Science and Technology Agency, Tokyo, Japan; 4 Department of Biotechnology and Life Science, Tokyo University of Agriculture and Technology 13125, Tokyo, Japan; Rochester Institute of Technology, College of Science-Dean's Office, Rochester, New York, USA

**Keywords:** PET hydrolase, compost metagenome, degenerate primers, amplicon sequencing

## Abstract

We report a collection of amplicon sequences of putative polyethylene terephthalate (PET) hydrolases from two different composts in Japan. Employing previously designed degenerate primers, we identified 31 and 22 sequences from industrial and agricultural composts, respectively, confirming the presence of highly homologous PET hydrolase genes across different compost environments.

## ANNOUNCEMENT

Enzymatic hydrolysis of polyethylene terephthalate (PET) is a key strategy for sustainable plastic recycling (1, 2). Recent studies have identified diverse PET hydrolases from various environments (3–7). Among these, composts containing thermophilic microorganisms are particularly rich sources of these enzymes (2, 8). Sonnendecker et al. designed degenerate primers targeting PET hydrolases, enabling efficient gene discovery (8). However, these primers have been applied to only a few compost samples from Germany, and their utility across geographically distant regions and different composting systems remains unexplored. In this study, we used these degenerate primers to collect PET hydrolase sequences from two compost sites in Japan: a high-temperature industrial compost (Compost 1) and an agricultural compost (Compost 2).

Compost 1 was collected from the Miyakojima City Resources Recycling Center in Okinawa (24°45′15.1″N 125°19′52.2″E) on 13 June 2016. Compost 2 was sampled from Kurkku Fields in Chiba (35°19′33.5″N 139°58′53.4″E) on 14 March 2024. DNA was extracted using ISOIL (Nippon Gene) for Compost 1 and the QIAamp PowerFecal Pro DNA Kit (QIAGEN) for Compost 2. Polymerase chain reaction (PCR) was performed using TaKaRa Ex Premier DNA Polymerase (Takara Bio) with degenerate primers (Fw: 5′-ATGGMSAACCCSTACGAGCGCGG-3′, Rev: 5′-GWRSGGGCAGKTGSMSCGGTACT-3′) (8). These primers were designed to amplify sequences encoding the signal-peptide-lacking (mature) forms of PET hydrolases (8). The resulting ~800–900 bp amplicons were excised from agarose gels using NucleoSpin Gel and PCR Clean-up (MACHEREY-NAGEL) and amplified using TaKaRa Ex Taq Hot Start Version (Takara Bio) with the same primer set. The amplified products were purified and cloned into the pTAC-1 vector using the FEW^Blue^ TA Cloning Kit (BioDynamics Laboratory). Inserts were amplified through colony PCR using KOD One PCR Master Mix (Toyobo) with vector-specific primers (Fw: 5′-GTAAAACGACGGCCAGT-3′, Rev: 5′-CAGGAAACAGCTATGAC-3′). Purified amplicons were Sanger sequenced. Homologous protein searches were performed for each deduced protein using BLASTP against the National Center for Biotechnology Information (NCBI) ClusteredNR (nr_cluster_seq) database. A phylogenetic tree was obtained using the neighbor-joining method with 1,000 bootstrap replicates in MEGA software (v12.1.2) (9, 10).

**Fig 1:**
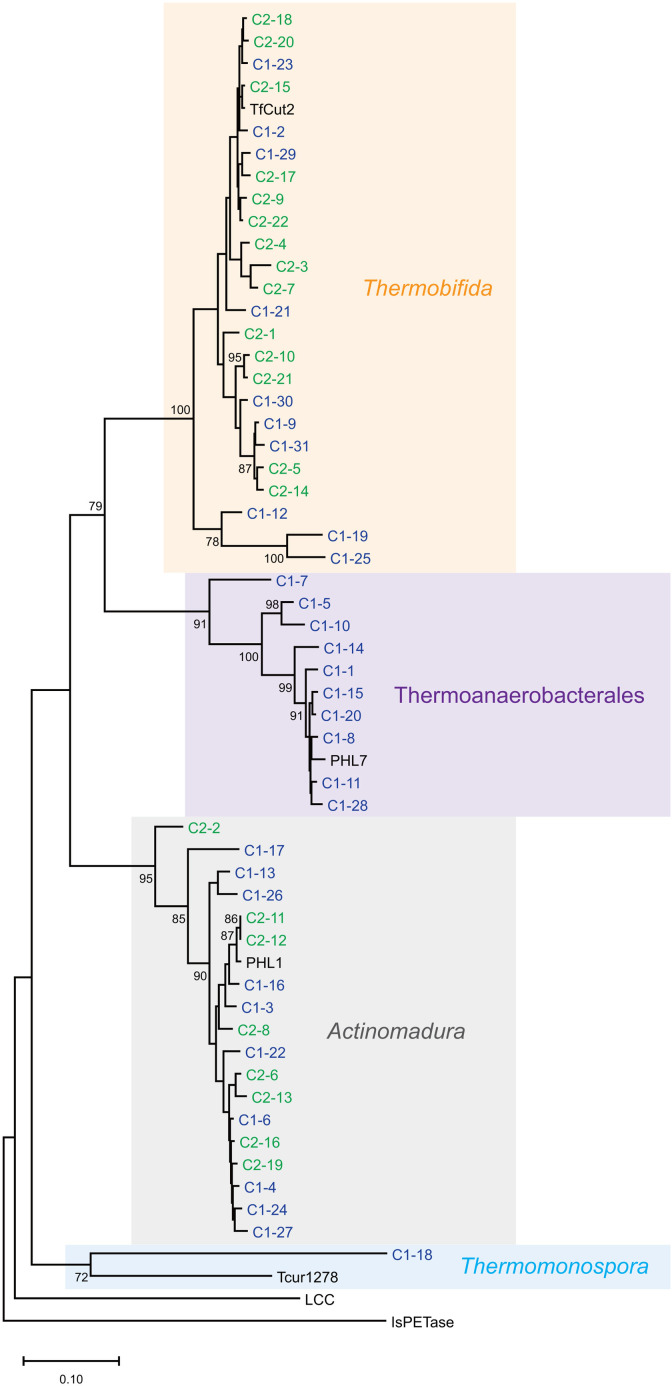
Neighbor-joining phylogenetic tree of 53 putative PET hydrolase sequences from two Japanese composts. Sequences from composts 1 (industrial compost) and 2 (agricultural compost) are shown in blue and green, respectively. Bootstrap values ≥ 70% (1,000 replicates) are indicated at branch points. Reference sequences are included to represent the major known lineages of PET hydrolases: cutinase from *Thermobifida fusca* (TfCut2; WP_011291330.1), PHL7 (SAY37592.1), PHL1 (SAY37579.1), cutinase from *Thermomonospora curvata* (Tcur1278; ACY96861.1), leaf-branch compost cutinase (LCC; AEV21261.1), and PETase from *Ideonella sakaiensis* (IsPETase; WP_054022242.1). The sequences of mature TfCut2, Tcur1278, LCC, and IsPETase were used. Major phylogenetic clusters are highlighted with background colors and labeled by taxonomic origin (*orange*, *Thermobifida*-derived enzymes; *purple*, Thermoanaerobacterales-derived enzymes; *gray*, *Actinomadura*-derived enzymes; *light blue*, *Thermomonospora*-derived enzymes). The clusters were defined based on the taxonomic affiliation of the most homologous proteins identified by BLASTP (Table 1) and supported by monophyletic grouping in the neighbor-joining tree. Scale bar indicates 0.10 substitutions per site.

From Compost 1, we identified 31 distinct sequences grouped into three major phylogenetic clusters with one additional sequence (Table 1 and Fig. 1). Ten sequences showed high identity to Thermoanaerobacterales-derived proteins and clustered with PHL7, a high-activity PET hydrolase (8). Another 10 sequences showed high identity to bis(hydroxyethyl) terephthalate hydrolases from *Actinomadura hallensis* and clustered with PHL1, a previously characterized PET hydrolase (8). Ten sequences were similar to *Thermobifida*-derived cutinases/PET hydrolases and clustered with a well-characterized PET hydrolase, TfCut2 (11–13). The remaining sequence showed similarity to a *Thermomonospora*-derived enzyme. From Compost 2, we obtained 22 sequences comprising two groups: sequences showing high identity to *Thermobifida*-derived cutinases/PET hydrolases (14/22) and bis(hydroxyethyl) terephthalate hydrolases from *A. hallensis* (8/22) (Table 1 and Fig. 1). All 53 deduced proteins possessed conserved residues, including the catalytic triad, aromatic clamp, and oxyanion hole, which are essential for PET hydrolytic activity (3) (see Data Availability). This study demonstrates the applicability of the degenerate primers to industrial and agricultural composts in Japan, confirming the presence of highly homologous PET hydrolase genes across different compost environments.

**TABLE 1:** Identification of coding sequences for putative PET hydrolases from composts 1 and 2^*a*^

Compost	#	Accession number	Most homologous protein	Query cover [%]	Identity [%]
1	C1-1	LC915630	Dienelactone hydrolase family protein [Thermoanaerobacterales bacterium] (MFO7281263.1)	100	98.06
	C1-2	LC915631	Cutinase 1, partial [*Thermobifida alba*] (ADV92525.1)	100	96.95
	C1-3	LC915632	Bis(hydroxyethyl) terephthalate hydrolase [*Actinomadura hallensis*] (WP_141974438.1)	99	96.90
	C1-4	LC915633	Bis(hydroxyethyl) terephthalate hydrolase [*Actinomadura hallensis*] (WP_141974438.1)	99	95.35
	C1-5	LC915634	Dienelactone hydrolase family protein [Thermoanaerobacterales bacterium] (MFO7281263.1)	99	91.41
	C1-6	LC915635	Bis(hydroxyethyl) terephthalate hydrolase [*Actinomadura hallensis*] (WP_141974438.1)	99	95.74
	C1-7	LC915636	Dienelactone hydrolase family protein [Thermoanaerobacterales bacterium] (MFO7281263.1)	99	84.17
	C1-8	LC915637	Dienelactone hydrolase family protein [Thermoanaerobacterales bacterium] (MFO7281263.1)	99	99.22
	C1-9	LC915638	Cutinase/poly(ethylene terephthalate) hydrolase [*Thermobifida fusca*] (WP_193587070.1)	99	99.23
	C1-10	LC915639	Dienelactone hydrolase family protein [Thermoanaerobacterales bacterium] (MFO7281263.1)	100	90.35
	C1-11	LC915640	Dienelactone hydrolase family protein [Thermoanaerobacterales bacterium] (MFO7281263.1)	99	98.83
	C1-12	LC915641	Cutinase/poly(ethylene terephthalate) hydrolase [*Thermobifida fusca*] (WP_193587070.1)	99	94.21
	C1-13	LC915642	Bis(hydroxyethyl) terephthalate hydrolase [*Actinomadura hallensis*] (WP_141974438.1)	99	94.21
	C1-14	LC915643	Dienelactone hydrolase family protein [Thermoanaerobacterales bacterium] (MFO7281263.1)	99	95.72
	C1-15	LC915644	Dienelactone hydrolase family protein [Thermoanaerobacterales bacterium] (MFO7281263.1)	100	98.84
	C1-16	LC915645	Bis(hydroxyethyl) terephthalate hydrolase [*Actinomadura hallensis*] (WP_141974438.1)	100	98.07
	C1-17	LC915646	Bis(hydroxyethyl) terephthalate hydrolase [*Actinomadura hallensis*] (WP_141974438.1)	100	91.89
	C1-18	LC915647	Poly(ethylene terephthalate) hydrolase family protein [uncultured *Thermomonospora* sp.] (WP_289009985.1)	100	98.46
	C1-19	LC915648	Chain A, Esterase [*Thermobifida alba*] (6AID_A)	100	97.70
	C1-20	LC915649	Chain A, Polyester Hydrolase Leipzig 7 (PHL-7), catalysis-deficient S131A mutant [unidentified] (8BRA_A)	100	97.30
	C1-21	LC915650	Cutinase 1, partial [*Thermobifida alba*] (ADV92525.1)	100	95.40
	C1-22	LC915651	Bis(hydroxyethyl) terephthalate hydrolase [*Actinomadura hallensis*] (WP_141974438.1)	99	93.39
	C1-23	LC915652	Cutinase 1, partial [*Thermobifida alba*] (ADV92525.1)	100	97.70
	C1-24	LC915653	Bis(hydroxyethyl) terephthalate hydrolase [*Actinomadura hallensis*] (WP_141974438.1)	99	94.96
	C1-25	LC915654	Cutinase/poly(ethylene terephthalate) hydrolase [*Thermobifida alba*] (WP_248591948.1)	100	97.70
	C1-26	LC915655	Bis(hydroxyethyl) terephthalate hydrolase [*Actinomadura hallensis*] (WP_141974438.1)	100	93.82
	C1-27	LC915656	Bis(hydroxyethyl) terephthalate hydrolase [*Actinomadura hallensis*] (WP_141974438.1)	99	94.57
	C1-28	LC915657	Chain A, Polyester Hydrolase Leipzig 7 (PHL-7), catalysis-deficient S131A mutant [unidentified] (8BRA_A)	100	97.30
	C1-29	LC915658	Cutinase 1, partial [*Thermobifida alba*] (ADV92525.1)	100	96.93
	C1-30	LC915659	Cutinase/poly(ethylene terephthalate) hydrolase [*Thermobifida fusca*] (WP_193587070.1)	99	97.31
	C1-31	LC915660	Cutinase/poly(ethylene terephthalate) hydrolase [*Thermobifida fusca*] (WP_193587070.1)	100	98.85
2	C2-1	LC915661	Cutinase 1, partial [*Thermobifida alba*] (ADV92525.1)	100	94.25
	C2-2	LC915662	Bis(hydroxyethyl) terephthalate hydrolase [*Actinomadura hallensis*] (WP_141974438.1)	100	90.04
	C2-3	LC915663	Cutinase/poly(ethylene terephthalate) hydrolase [*Thermobifida fusca*] (WP_193587070.1)	100	97.32
	C2-4	LC915664	Cutinase 1, partial [*Thermobifida alba*] (ADV92525.1)	100	95.40
	C2-5	LC915665	Cutinase/poly(ethylene terephthalate) hydrolase [*Thermobifida fusca*] (WP_193587070.1)	99	100
	C2-6	LC915666	Bis(hydroxyethyl) terephthalate hydrolase [*Actinomadura hallensis*] (WP_141974438.1)	99	96.50
	C2-7	LC915667	Cutinase 1, partial [*Thermobifida alba*] (ADV92525.1)	100	94.27
	C2-8	LC915668	Bis(hydroxyethyl) terephthalate hydrolase [*Actinomadura hallensis*] (WP_141974438.1)	99	97.67
	C2-9	LC915669	Cutinase 1, partial [*Thermobifida alba*] (ADV92525.1)	100	96.93
	C2-10	LC915670	Cutinase/poly(ethylene terephthalate) hydrolase [*Thermobifida fusca*] (WP_193587070.1)	100	96.17
	C2-11	LC915671	Bis(hydroxyethyl) terephthalate hydrolase [*Actinomadura hallensis*] (WP_141974438.1)	99	98.84
	C2-12	LC915672	Bis(hydroxyethyl) terephthalate hydrolase [*Actinomadura hallensis*] (WP_141974438.1)	99	98.84
	C2-13	LC915673	Bis(hydroxyethyl) terephthalate hydrolase [*Actinomadura hallensis*] (WP_141974438.1)	99	96.51
	C2-14	LC915674	Cutinase/poly(ethylene terephthalate) hydrolase [*Thermobifida fusca*] (WP_193587070.1)	99	99.23
	C2-15	LC915675	Cutinase 1, partial [*Thermobifida alba*] (ADV92525.1)	100	98.09
	C2-16	LC915676	Bis(hydroxyethyl) terephthalate hydrolase [*Actinomadura hallensis*] (WP_141974438.1)	99	94.94
	C2-17	LC915677	Cutinase 1, partial [*Thermobifida alba*] (ADV92525.1)	100	96.95
	C2-18	LC915678	Cutinase 1, partial [*Thermobifida alba*] (ADV92525.1)	100	97.71
	C2-19	LC915679	Bis(hydroxyethyl) terephthalate hydrolase [*Actinomadura hallensis*] (WP_141974438.1)	99	95.33
	C2-20	LC915680	Cutinase 1, partial [*Thermobifida alba*] (ADV92525.1)	100	96.95
	C2-21	LC915681	Cutinase/poly(ethylene terephthalate) hydrolase [*Thermobifida fusca*] (WP_193587070.1)	100	96.17
	C2-22	LC915682	Cutinase 1, partial [*Thermobifida alba*] (ADV92525.1)	100	97.71

^*a*^ Homologous protein searches were performed for each deduced protein using BLASTP against the NCBI ClusteredNR (nr_cluster_seq) database (https://blast.ncbi.nlm.nih.gov/Blast.cgi).

## Data Availability

The sequence data have been deposited in DDBJ/ENA/GenBank under accession numbers LC915630–LC915682. These are associated with BioProject PRJDB40222 and BioSample SAMD01813938 and SAMD01813939. Detailed methods are available on protocols.io (https://dx.doi.org/10.17504/protocols.io.x54v9n9opl3e/v1). Multiple sequence alignment of the deduced amino acid sequences of the 53 amplicons with four reference PET hydrolases (LCC, TfCut2, PHL7, and PHL1) is available at Figshare (https://doi.org/10.6084/m9.figshare.31605196).
